# Trastuzumab and docetaxel in a preclinical organotypic breast cancer model using tissue slices from mammary fat pad: Translational relevance

**DOI:** 10.3892/or.2015.4074

**Published:** 2015-06-24

**Authors:** LOREDANA VESCI, VALERIA CAROLLO, GIUSEPPE ROSCILLI, LUIGI AURISICCHIO, FABIANA FOSCA FERRARA, LUIGI SPAGNOLI, RITA DE SANTIS

**Affiliations:** 1 Biotechnology, Research and Development, Sigma-Tau Industrie Farmaceutiche Riunite S.p.A., I-00040 Pomezia, Italy; 2 Takis Biotechnology, Castel Romano, I-00128 Rome, Italy; 3 Tissue Macro Array Lab, university of Tor Vergata, I-00133 Rome, Italy

**Keywords:** tissue slices, breast cancer, mammary fat pad, trastuzumab, docetaxel

## Abstract

With the ever-increasing number of drugs approved to treat cancers, selection of the optimal treatment regimen for an individual patient is challenging. Breast cancer complexity requires novel predictive methods and tools. In the present study, we set up experimental conditions to obtain an '*ex vivo*' organotypic culture from xenotransplanted mice aiming at recapitulating the human clinical condition. The effect of trastuzumab (large biological molecule) and docetaxel (small chemical entity) was subsequently investigated on this organotypic model and compared with *in vivo* and *in vitro* activity on tumor cells. Tissue slices of 200 *µ*m were obtained from mammary fat pad of SCID mice xenotransplanted with human MCF-7 breast cancer cells. Viability and proliferation were evaluated by 3-(4,5-dimethylthiazol-2-yl)-2,5-diphenyltetrazolium bromide (MTT) colorimetric assay and Ki-67 immunohistochemistry,and apoptosis by cleaved caspase-3 immunohistochemistry. *In vivo* antitumor activity of trastuzumab and docetaxel was determined by caliper measurement of tumor volume and Ki-67 expression on explanted masses by immunohistochemistry. A Teflon support and normoxia were necessary experimental conditions to obtain high viability of excised breast cancer infiltrated mammary fat pad slices upon 48 h cultivation, as shown by MTT proliferation assay, and Ki-67 expression. Breast cancer tissue slices treated for 48 h with trastuzumab or docetaxel showed a significant dose-dependent reduction of viability by MTT assay. Consistently, both drugs down-modulated Ki-67 and increased cleaved caspase-3. Tumor masses collected from docetaxel-or trastuzumab-treated mice showed a similar reduction of proliferation markers. By contrast, MCF-7 cell cultures were significantly inhibited by docetaxel but not by trastuzumab. Tumor tissue slices represent a more predictive experimental cancer model compared to cell cultures for both small and large molecule antitumor efficacy. This observation supports the relevance of microenvironment in the overall tumor biology and response to therapeutics.

## Introduction

Breast cancer is the most common type of cancer in the world with more than 1.3 million patients and a mortality rate of ~450,000 deaths/year (1). The high incidence of disease is suggestive of slow progress in the prevention programs. In the treatment of women with localized disease, mortality rates have been improved, but the median survival time in the metastatic setting is only 25 months (2). Systemic chemotherapy as treatment of choice for most cancer types and its relatively modest improvement in survival associated with significant toxicity, highlighted the need over the past decade to develop targeted therapies. Targeted drugs can restore the deregulated signalling transduction pathway in which a mutated gene and its encoded protein is involved (3). Targeted therapy represents the major hope against cancer and a substantial step towards personalized medicine, since this type of drugs is mostly active in cancer cells without affecting normal healthy cells. However, the efficacy of these single signal transduction pathway inhibitors is in most cases modest, thus the progress against cancer is slow and it is translated into a few weeks of survival prolongation. Elucidation of the mechanisms beyond intrinsic and acquired tumor resistance to therapies is hampered by high inter-patient and intratumor heterogeneity (4–7). Consequently, a patient-specifc experimental approach is needed to analyze individual responses to therapies. Based on comprehensive gene expression profiling, breast tumors are classified into at least three major subtypes: luminal, human epidermal growth factor receptor 2^+^ (HER2^+^) and basal-like (8,9), with different risk factors for incidence, response to treatment, disease progression and preferential organ sites of metastases (10–12). Antitumor drugs, presently used in the clinical practice, have been previously characterized in the two-dimensional *in vitro* culture and *in vivo* xenograft tumor models. A broad analysis on investigational drugs, done at the National Cancer Institute, pointed out at a poor correlation between preclinical data from *in vitro* models and tumor xenografts and phase II efficacy data leading to the conclusion that only compounds that are successful in a large number of different models are likely to be effective in the clinic (13). Thus, frequent failures in drug development can be explained by the fact that the existing preclinical models do not represent the complexity (heterogeneity) that is typical of human tumors. We have, therefore, recently explored tissue slice technology via the Krumdieck tissue slicer, by preserving the tissues in the three-dimensional structure, it allows the setting up of a powerful and representative *ex vivo* tumor model. Moreover, taking into account the notion that cancer cell lines, passaged *in vitro* for years, may not reflect the biology of *in vivo* tumors, we compared the activity of docetaxel (small molecule) and trastuzumab (large molecule), both commonly used for breast cancer therapy, in cancer cell lines and organotypic tissue slices.

## Materials and methods

### Materials

The primary antibodies used in the present study were monoclonal rabbit anti-human Ki-67 antigen (NB600-1252; Novus Biologicals, Littleton, CO, USA) and cleaved caspase-3 (#9664; Cell Signaling Technology, Danvers, MA, USA). The secondary antibody was biotinylated goat anti-rabbit (#E0432; Dako, Denmark).

### Ethics statement

All studies were performed in accordance with the 'Directive 2010/63/UE' on the protection of animals used for scientific purposes, made effective in Italy by the Legislative Decree 4 March 2014, n. 26, and applying the principles of 3Rs (i.e., to replace, reduce and refine). Mice were purchased from Harlan Laboratories (Udine, Italy). All procedures performed on the animals were approved by the Animal Welfare Body and authorized by the Italian Ministry of Health, 46/2014-PR. At the end of the treatment period and before necropsy, mice were euthanized by compressed CO_2_ gas in a cylinder as indicated in the American Veterinary Medical Association (AVMA) Panel on Euthanasia according to the 1998 UKCCCR Guidelines for the Welfare of Animals in Experimental Neoplasia.

### Tumor xenograft in mammary fat pad

MCF-7 breast tumor cells were injected into the abdominal fat pad of SCID Beige (7×10^6^ cells/100 *µ*l/mouse), 24 h after the subcutaneous implantation of estrogen pellets containing 17β-estradiol (0.72 mg/pellet, 60-day release/mouse) (#SE-121; Innovative Research of America, Sarasota, FL, USA). Tumor lesions were measured with a Vernier caliper twice a week to reach a volume of around 300 mm^3^ prior to collection for tissue slice preparation.

For *in vivo* drug efficacy evaluation, tumor-bearing mice were also treated with docetaxel (Sigma-Aldrich, St. Louis, MO, USA) at 15 mg/10 ml/kg i.p. (q7dx3, once a week for 3 weeks) and trastuzumab at 10 mg/10 ml/kg i.p. (q7dx3) (Herceptin; Roche S.p.A., Milan, Italy). Tumor lesions were measured with a Vernier caliper twice a week to reach a volume of around 300 mm^3^ before to be collected for analysis of marker proliferation.

### Tumor explantation

Mice were sacrificed, tumors explanted and immediately cut using the Vibratome VT1200 (Leica, Germany) to obtain 200 *µ*M thick slices of the whole tumors.

### Tissue slice maintaining conditions

To set the best experimental conditions, tumor slices were maintained in floating normoxic conditions for a maximum of 4 days using a modular incubator chamber 37°C in 6-multiwell plates, using Dulbecco's modified Eagle's medium (DMEM) with 10% FBS, a mix of penicillin-streptomycin and 1% L-glutamine as culture medium. Medium change was performed every 24 h.

Subsequently, Teflon supporting and normoxia were evaluated. Tumor slices were maintained at 37°C and 5% CO_2_ (normoxic conditions) for a maximum of 4 days on organotypic Teflon inserts (#PICM01250 MilliCell; Millipore, Billerica, MA, USA) in 6-multiwell plates, using DMEM (#D5921; Sigma-Aldrich) with 10% FBS, 1% L-glutamine, a mix of penicillin-streptomycin as culture medium. Medium change was performed every 24 h.

### Cell viability determination in tissue slices

The viability of tumor slices was evaluated using the 3-(4,5-dimethylthiazol-2-yl)-2,5-diphenyltetrazolium bromide (MTT) (#M5655; Sigma-Aldrich) under normoxia (21% O_2_) after a maximum of 3 days of incubation. Moreover, the integrity of the slices was assessed by hematoxylin and eosin (H&E) staining.

### Immunohistochemistry

Immunochemistry analysis was performed by employing paraffin sections. Tissue slices of 200 *µ*m were paraffin-embedded (horizontal orientation). The sections were incubated with primary antibodies overnight. After washing, secondary antibodies were applied at 1:200 dilutions for 30 min. Images were acquired using a Nikon microscope (Eclipse 80i, Nikon, Japan) with a Nikon digital camera (DXM1200F). H&E staining was performed to examine the extent of the slice integrity.

### Drug treatment of slice cultures

Slices obtained from three mice were evaluated in triplicate for MTT assay 2 or 3 days after slicing. For treatment of slices, docetaxel (Sigma-Aldrich) and trastuzumab (Herceptin) were used. The concentrations were chosen on the basis of results obtained from tumor cell growth inhibition assay. Before testing docetaxel in its final concentrations of 200-20 nM, it was dissolved in dimethyl sulfoxide (DMSO) and diluted in culture medium. Trastuzumab was tested at the concentrations of 20-0.02 *µ*g/ml. The incubation period for treatment was up to 2 days. At the end of the treatment, tissue slices were incubated with 5 mg/ml of MTT at 37°C for 1 h, harvested and precipitated-salt extracted by incubation with 0.1 M HCl-isopropyl alcohol at room temperature for 40 min. Viability values were determined by dividing the optical density of the formazan at 570 nm by the dry weight of the explants.

### Growth inhibition tumor cell assay

MTT assay was performed for evaluation of the cell viability within 3 days of culture. Tumor cells were seeded 3,000 cells/well. upon 24 h, tumor cells were treated with different concentrations of trastuzumab (200-20 *µ*g/ml) and docetaxel (500-0.5 nM) for 3 days. At the end of treatment, the medium was discarded, cells were washed twice with PBS, and replaced by MTT-containing medium. The plates were incubated at 37°C for 4 h. Then the MTT solution was discarded and without washing, DMSO was added to dissolve the formazan formed. After 15 min incubation, cell plates were transferred to the microplate reader (Victor, Wallac) and the absorbance at 570 nm was measured. The results were expressed as percent of drug-treated viable cells in comparison with vehicle-treated cells. The IC_50_ values were calculated using four-parameter fit by ALLFIT program.

### Statistical analysis

All data are expressed as the mean value ± standard error (SE) and differences between groups were analyzed using Mann-Whitney U test. Mean values are considered significantly different at P<0.05.

## Results

### In vitro and in vivo antitumor activity of docetaxel and trastuzumab

**Figure 1:**
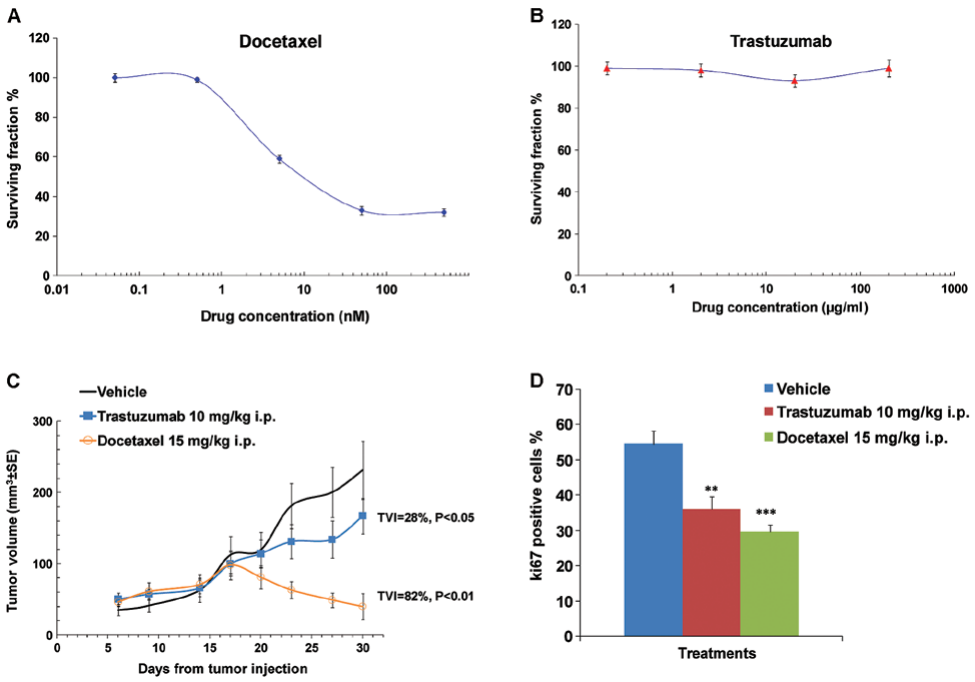
*In vitro* and *in vivo* antitumor activity of docetaxel and trastuzumab on MCF-7 breast carcinoma. (A and B) *In vitro* MCF-7 tumor cell growth inhibition induced by docetaxel and trastuzumab evaluated after 3 days of culture. Cell survival was evaluated by MTT assay. Data are means of two experiments and each point was in triplicate. The IC_50_ values expressed as mean ± SE were 7±0.05 nM for docetaxel and >200 *µ*g/ml for trastuzumab. (C) MCF-7 mammary fat pad tumor-bearing mice were treated as indicated according to the schedule q7dx3 (n=8/group), starting 6 days after tumor implantation. TVI was evaluated 10 days after the last treatment. The statistical comparison of drug-treated group vs. the vehicle treated group was performed by Mann-Whitney U test. (D) Percent of Ki-67^+^ cells, as detected by immunohistochemical analysis in mammary fat pad tumor lesions of mice treated as indicated (8 mice/group) (^**^P<0.01 and ^***^P<0.001 vs. vehicle-treated group, Mann-Whitney U test).

In order to evaluate the *in vitro* and *in vivo* antitumor activity of docetaxel and trastuzumab, standard human MCF-7 cell culture and a tumor xenograft study in nude mice were carried out. These tumor cells were first of all analysed for ERBB2 membrane expression by FACS analysis with 97.6% of tumor cells positive for ERBB2-conjugated antibody (R&S, FAB 11299) (data not shown). Tumor cell proliferation was tested by MTT assay after 3 days of culture showing a dose-dependent inhibitory effect of docetaxel but not trastuzumab, resulting in an IC_50_ of 7±0.1 nM and >200 *µ*g/ml, respectively (Fig. 1A and B). Consistently with *in vitro* data, docetaxel strongly inhibited the growth of the MCF-7 xenografts (TVI=82%; P<0.01) whereas trastuzumab exhibited a lower but significant antitumor activity (TVI=28%; P<0.05) (Fig. 1C). Reduction of Ki-67 expression correlated with both docetaxel-dependent and trastuzumab-dependent antitumor activity (Fig. 1D). Ki-67 is an important marker predicting recurrence, prognosis and overall survival in breast cancer patients (14–16). It has also been associated with positive axillary lymph nodes in most studies (17,18). Recently, Ki-67 was integrated as a prognostic factor into molecular typing in prognosis of patients with luminal B breast cancer (19). Therefore, we investigated Ki-67 expression in further tumor slice experiments.

### Tumor slice preparation

**Figure 2:**
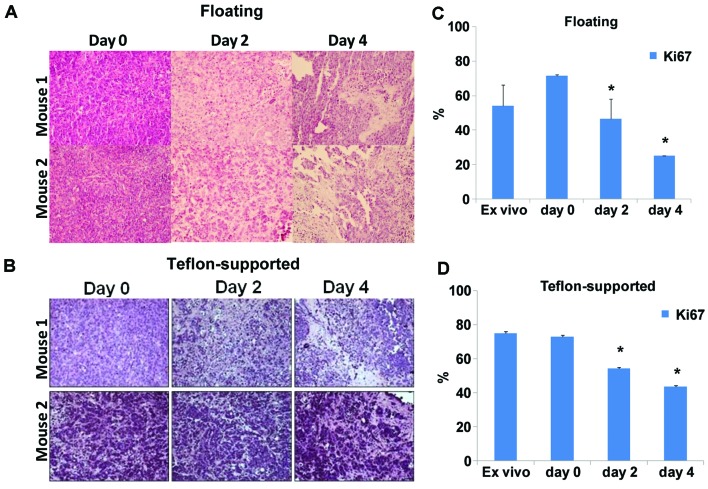
Tissue integrity of tissue slices in floating and teflon supported conditions. (A and B) Tissue slices were processed immediately or kept for 2 or 4 days in culture medium, floating or Tefon-supported conditions, then fixed in formalin and paraffin-embedded for analysis of H&E staining. Images were from two representative mice and were digitally scanned ×20 (AlexaSoft X-PRO). (C and D) Percentage of Ki-67 as detected by immunohistochemical analysis in mammary fat pad of tumor implanted samples (*ex vivo*) and tissue slices after 0, 2 or 4 days of culture in floating or Teflon-supported conditions. The data are means from 3 mice in triplicate (^*^P<0.05 vs. tissue slice on day 0, Mann-Whitney U test). H&E, hetoxylin and eosin.

The set up of *ex vivo* organotypic cultures of human breast carcinoma was carried out before drug testing. Tumor slices of 200 *µ*m were obtained from MCF-7 xenotransplanted mammary fat pads by the use of a vibratome. Slices were subjected to histological analysis to identify the best experimental conditions to preserve the tissue culture viability. The slices were maintened in normoxia for 2 or 4 days in floating or in Teflon-supported conditions before analyses. H&E staining of tumor slices revealed a high qualitative viability in the Teflon-supported compared to floating condition (Fig. 2A and B). Accordingly, significantly decreased expression of Ki-67 was found by immunohistochemistry in floating slices compared to Teflon-supported slices (Fig. 2C and D).

These results indicated that an inert support is useful to keep slices viable and tumor cells in a proliferative status, thus indicating that tissue slices represent a physiologically more relevant model compared to tissue cultures to study solid cancer. Moreover, despite the lack of blood supply the viability of Teflon-supported 200 *µ*m thick tissue slices, maintained in normoxia, suggests an adequate nutrient diffusion from the culture medium.

**Figure 3:**
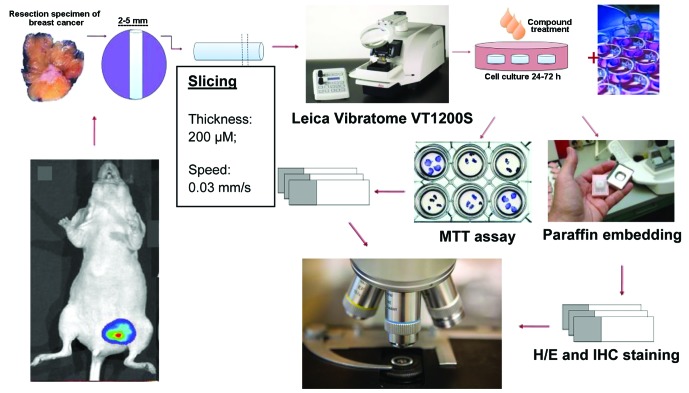
Representative scheme of tissue slice production and analysis.

A schematic representation of the procedure to obtain organotypic breast cancer tissue slices from MCF-7 mammary fat pad xenotransplant is shown in Fig. 3.

### Evaluation of docetaxel and trastuzumab antitumor activity on breast cancer tissue slices

**Figure 4:**
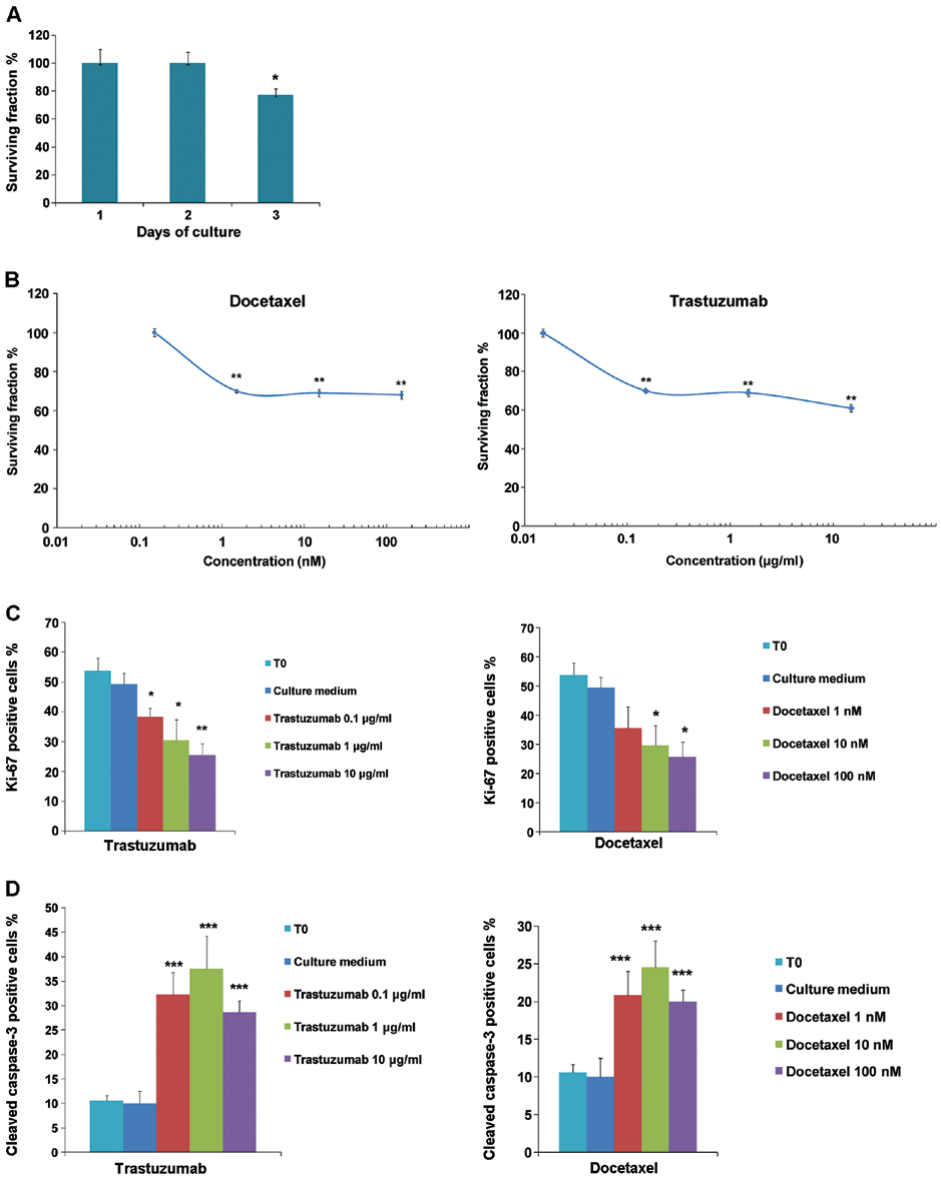
Viability of tissue slices on Teflon support upon docetaxel or trastuzumab treatment. (A) MTT assay was carried out on tissue slices after 1–3 days in culture. Data are mean ± SE in triplicate of 3 slices/time-point. (^*^P<0.05 vs. day 1 Mann-Whitney U test). (B) Teflon-supported tissue slices treated with trastuzumab or docetaxel for 2 days. Each point is the mean ± SE of triplicate slices (^**^P<0.01 vs. culture medium, Mann-Whitney U test). (C and D) Percentage of Ki-67 and cleaved caspase-3 positive cells of tissue slices processed immediately (t0) or kept 2 days in culture medium with or without indicated drugs. Data were mean ± SE from two tissue slices untreated and drug-treated (^*^P<0.05, ^**^P<0.01 and ^***^P<0.001 vs. tissue slice on day 0, Mann-Whitney U test).

**Figure 5:**
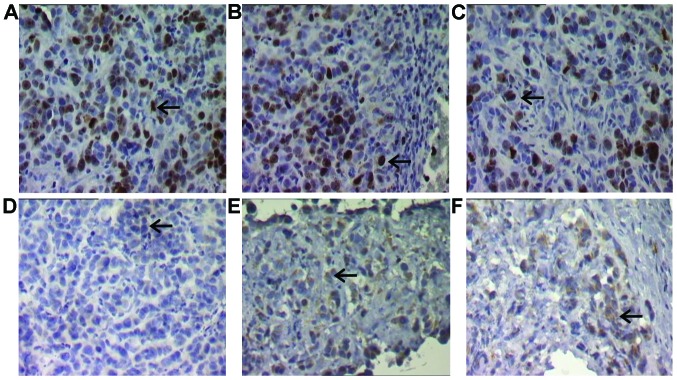
Immunohistochemistry of tissue slices treated with trastuzumab and docetaxel. (Ki-67, arrows; A-C) and (cleaved caspase-3, arrows; D-F) were quantified by counting the number of positively stained cells in 4 fields of view at x40. Images are representative of untreated (A, D), trastuzumab at 100 *µ*g/ml (B and E) and docetaxel at 100 nM (C and F). Images were digitally scanned ×40 (AlexaSoft X-PRO).

Because substantial morphological integrity of tissues, defined as preservation of general architecture including epithelial structures and their spatial relationship to stroma, was observed in tumor tissue slices up to 2 days of culture (Fig. 2). MTT assay was performed to quantify viability. Data in Fig. 4A show similar number of viable cells after 1 or 2 days dropping to ~80% after 3 days of cultivation. Further experiments were then performed cultivating the tissue slices for 2 days in the presence of different concentrations of docetaxel or trastuzumab. MTT assay revealed a significant dose-dependent loss of viable cells upon treatment with both drugs (Mann-Whitney U test; ^**^P<0.01) (Fig. 4B). In agreement with this result, the percentage of proliferating cells assessed by Ki-67 immunostaining significantly decreased upon docetaxel or trastuzumab exposure (Fig. 4C), paralleled by increased percentage of cleaved caspase-3 positive cells (Fig. 4D). In particular, biomarkers of proliferation and apoptosis used to quantify effects of trastuzumab and docetaxel were evaluated by counting numbers of positively stained cells (Fig. 5A–F). Taken together, these data show that tissue slices can better refect the *in vivo* experimental outcome.

## Discussion

In the present study we report a method to obtain organotypic breast cancer tissue slices useful to evaluate the efficacy of both small and a large molecule drugs. The advantage of the tissue slice cultures is that the complex cross-talk between matrix and cells as well as the cellular heterogeneity and susceptibility to drugs is preserved avoiding potential source of artefacts deriving from isolation of tumor cells from their biological environment (20,21). Moreover, the presence ofb ECM provides important differentiation cues, for example, by signalling via Toll-like receptors and integrins (22–24), thus further supporting the use of tissue slice method for screening of therapeutics. Organotypic tissue slice cultures offer a more attractive option compared to cell line cultures or subcutaneous xenografts both lacking the complexity of a tumor nested in a mammary gland (25–27). Tissue slice samples with a thickness between 400–800 *µ*m, cultured 24 h were previously used to evaluate the transcriptional effects of 1,25(OH)_2_D_3_ in breast cancer benefitting from the heterogeneous combination of epithelial and stromal cells that secrete a variety factors affecting the overall response of tumor cells to the vitamin (28). Tissue slices of 250 *µ*m from human primary invasive ductal breast tumors were also employed to test tamoxifen and doxorubicin activity, confirming the complexity and heterogeneity of breast cancer among patients (29). Primary tissue slices of 400 *µ*m were cultured in the presence of rapamycin, showing that such a culture method preserves the tumor AKT/mTOR pathway activity (30). Moreover, slice cultures of 350 *µ*m from head and neck squamous cell carcinoma from patients were also used to test cisplatin, docetaxel and cetuximab allowing the design of personalized therapies and investigation of mechanisms of tumor resistance (31,32). It is becoming increasingly evident that the development of cancer and the response to anti-cancer drugs not only depend on genetic alterations but also on specific interactions between tumor cells and surrounding tissue components. In invasive breast carcinoma, differentiated myoepithelial cells and intact basement membranes are lost and tumor cells are in direct contact with an activated collagenous tumor stroma (27). To simulate such conditions either three-dimensional tissue cultures using several biomatrices or co-culture experiments with tumor fibroblasts have been performed. However, these systems cannot mimic the complex tissue architecture and the high degree of variability seen in individual tumors. Therefore, to evaluate the activity of docetaxel and trastuzumab antitumor drugs we used tissue slice cultures starting from orthotopic human breast cancer xenografts in mice and compared results with *in vivo* efficacy. Our data support the use of the patient's tissue slices as predictive translational model for personalized therapies.

So far, neither *in vitro* nor *ex vivo* testing technologies have gained a significant impact in predicting clinical efficacy of therapeutic treatments. Present data can be framed within a widespread effort to identify testing conditions useful to increase the success rate of drug investigation, thus facilitating translational medicine.

Here we provide evidence that breast cancer tissue slice is a more useful model than cell cultures to predict antitumor efficacy of both small and large molecules. Sensitivity of breast tumors to anticancer drugs depends upon dynamic interactions between epithelial tumor cells and their microenvironment including stromal cells and extracellular matrix. Moreover, in addition to cancer cell viability, this model improves the understanding of the impact of a given treatment on stromal and endothelial cells and the distribution of a biologic, a small molecule or viral vectors within tumor microenvironment. Another potential application may include the *ex vivo* addition of immune cells for OncoImmunology studies.

In conclusion, tissue slices represent an optimal tool to investigate therapeutics and further studies to evaluate this model for novel investigational drugs are warranted.
